# Metagenomic analysis suggests that tomato root-knot nematode infestation disrupts rhizosphere microbial networks, consistent with reduced disease suppression

**DOI:** 10.3389/fmicb.2026.1798902

**Published:** 2026-05-04

**Authors:** Jie Duan, Yang Chen, Xiaojing Zhang, Chenchen Li, Tianmei Gao, Kemei Li

**Affiliations:** Key Laboratory of the Pest Monitoring and Safety Control of Crops and Forests of the Xinjiang Uygur Autonomous Region/Key Laboratory of Prevention and Control of Invasive Alien Species in Agriculture and Forestry of the North-western Desert Oasis (Co-construction by Ministry and Province), College of Agronomy, Ministry of Agriculture and Rural Affairs, Xinjiang Agricultural University, Urumqi, China

**Keywords:** functional redundancy, metagenomics, microbial interaction network, network stability, rhizosphere microbiome, root-knot nematode

## Abstract

**Introduction:**

The rhizosphere microbiome serves as a critical line of defense for plant health and soil-borne disease suppression. However, the underlying mechanisms by which root-knot nematodes (RKN), a devastating soil-borne pathogen, undermine putative disease-suppressive function through destabilizing microbial interaction networks remain poorly understood.

**Methods:**

This study employed metagenomic sequencing coupled with microbial co-occurrence network analysis to systematically compare the community structure, interaction network topology, and functional gene profiles of the rhizosphere microbiome between healthy and RKN-infected tomato plants.

**Results:**

Our findings revealed that RKN infection significantly altered the community structure of bacteria, fungi, and viruses. This disturbance was associated with a systematic simplification and loss of modularity within microbial interaction networks. Specifically, intra-domain bacterial networks exhibited reduced scale and connectivity, whereas fungal networks showed strengthened internal cohesion. Cross-kingdom interactions (e.g., bacteria-fungi) were severely weakened, resulting in a topological imbalance characterized by “tight within domains, loose between domains.” Functional profiling further indicated a distinct metabolic reprogramming in the infected rhizosphere, with a shift in resource allocation from growth and biosynthesis toward core energy acquisition and stress response.

**Discussion:**

Collectively, our results suggest that the putative decline in disease-suppressive function following RKN infection may be mechanistically rooted in the destabilization of microbial cooperative networks and the consequent loss of functional redundancy. This study provides a novel network-level ecological framework for understanding plant-microbe-pathogen interactions and lays a theoretical foundation for microbiome-based ecological management strategies against soil-borne diseases.

## 1 Introduction

The rhizosphere microbiome, often regarded as the plant’s “second genome,” is indispensable for maintaining host health and suppressing soil-borne diseases (Berendsen et al., 2012; Mendes et al., 2011; Trivedi et al., 2020). A stable, disease-suppressive rhizosphere microbiome exerts its disease-mitigating functions via two core mechanisms: niche competition and induced systemic resistance (ISR) (Li et al., 2021; Pieterse et al., 2014). A sophisticated hallmark of such host-mediated regulatory processes is the active recruitment of beneficial microorganisms; specifically, plants under pathogen challenge remodel their root exudate profiles to selectively enrich specific protective taxa (e.g., *Flavobacterium* spp.), thereby mounting a tailored, adaptive defense response (Andargie et al., 2023; Chen et al., 2020; Rudrappa et al., 2008). Root-knot nematodes (RKNs), as devastating sedentary endoparasites of plants, not only inflict direct physical damage on host roots but also drastically alter the composition, structure, and functional traits of the rhizosphere microbiome (Jones et al., 2013; La et al., 2024; Topalović et al., 2020). Intriguingly, accumulating evidence suggests that RKNs can trigger metabolic reprogramming and pre-activate parasitic effectors prior to physical invasion, solely in response to chemical signals from root exudates, thereby pre-conditioning the rhizosphere microenvironment to facilitate subsequent colonization (Wu et al., 2026; Zhao et al., 2019).

While recent studies have documented taxonomic shifts in the rhizosphere microbiome under RKN infestation (Cao et al., 2022; Song et al., 2023), the mechanistic basis by which RKN reshapes microbial interaction networks remains poorly understood at a systematic, ecological level. The stability and function of microbial communities are tightly dependent on complex interspecific interaction networks, whose topological features (e.g., connectivity, modularity) serve as key metrics for assessing community resilience and functional redundancy (Faust and Raes, 2012; Hernandez et al., 2021). Emerging evidence, however, indicates that RKN is not merely a passive disruptor but can actively manipulate and exploit these networks (Elhady et al., 2021; Yergaliyev et al., 2020). For instance, defensive compounds in maize root exudates can be metabolized by specific rhizosphere bacteria (e.g., *Pseudomonas*), which are then converted into nematode-attracting volatile signals, thereby facilitating the precise host localization of RKN (Song et al., 2023; Yergaliyev et al., 2020). Meanwhile, RKN infestation itself drives deterministic succession of both endophytic and rhizosphere microbial communities, accompanied by the enrichment of genes associated with key ecological functions (e.g., nitrogen fixation) (Li et al., 2023). Such bidirectional interactions profoundly modulate the dynamics of microbial networks: empirical studies have suggested that RKN infestation alters microbial coexistence patterns and may induce simplification or reconfiguration of network architecture (Huang et al., 2020, 2025; Lu et al., 2023; Zhou et al., 2019). Therefore, dissecting how RKN actively rewires the structure, stability, and co-occurrence patterns of microbial association networks—rather than merely observing network collapse—is of critical significance for uncovering the fundamental causes of the loss of disease-suppressive functions in the rhizosphere, and further developing targeted RKN management strategies via microbial community modulation (e.g., construction of functional synthetic microbial consortia) (Edwards et al., 2015; Lareen et al., 2016; Van Elsas et al., 2012).

Furthermore, metagenomic studies reveal that pathogen stress triggers a rapid functional reprogramming of the rhizosphere microbiome, often upregulating genes related to energy metabolism and nutrient cycling (Zhalnina et al., 2018). However, the ecological interpretation of these functional shifts remains contentious. Are they a metabolic cost incurred by the stressed plant (e.g., due to increased exudation enriching saprophytic functions) (Chaparro et al., 2014), or a synergistic strategy driven by the pathogen to create a favorable microenvironment (Liu et al., 2023)? Recent frameworks propose moving beyond the binary plant-pathogen view to a tripartite plant-resident microbiota-pathogen model, where resident microbes can play pivotal roles in either suppressing or, conversely, facilitating pathogen infection (Berendsen et al., 2012; Gui et al., 2025; Han et al., 2025). This “chemo-epigenetic” perspective underscores the complexity of interpreting functional changes (Gui et al., 2025). Critically, recent evidence that root exudates can exert sustained effects on microbiome function, including the resistome (Guan et al., 2025; Tan et al., 2025), highlights the long-term ecological consequences of such stress-induced reprogramming. Therefore, clarifying this “cost versus synergy” dilemma is essential for accurately modeling tripartite interactions and has practical implications for sustainable management, as root exudate components have been shown to have sustained effects on microbiome function, including the resistome.

Consequently, a systems biology approach that integrates taxonomic, interactional, and functional dimensions is urgently needed to decipher the rhizosphere microbiome’s ecological strategy under biotic stress (Berendsen et al., 2012). In this study, we applied metagenomic sequencing to comparatively analyze the rhizosphere microbiomes of healthy and RKN-infected crops. Our objectives were to: (1) characterize the impact of RKN infection on the community structures of bacteria, fungi, archaea, and viruses; (2) decode changes in the topology and stability of intra- and inter-kingdom microbial co-occurrence networks; (3) elucidate the reprogramming of community-wide functional profiles and key metabolic pathways; and (4) identify core taxa and functional modules in the stress response by constructing integrated microbe-gene co-functional networks. This study aims to address two central questions: Does RKN infection disrupt the stability of microbial ecological networks, thereby eroding the foundation of rhizosphere-mediated disease suppression? Furthermore, are the enriched functional modules under disease stress more reflective of a plant immune cost or a pathogen-driven strategy, potentially mediated through altered plant signaling that affects subsequent pathogen decisions? Our findings are expected to provide novel ecological insights into plant-microbe-pathogen systems and lay a theoretical foundation for developing microbiome-based ecological management strategies.

## 2 Materials and methods

### 2.1 Experimental design and sample collection

Root samples together with adhering rhizosphere soil were collected from healthy and root-knot nematode (RKN)-infected tomato plants (*Solanum lycopersicum* cv. “Moneymaker”) grown under greenhouse conditions in Korla, Xinjiang.

#### 2.1.1 Rhizosphere soil collection

Two groups were established: rhizosphere soil from RKN-infected plants (IRS) and from healthy plants (HRS). Samples were collected from tomato plants at the mature stage. RKN infection was confirmed by the presence of root galls on infected plants, while healthy plants showed no symptoms. Both groups were sampled at the same developmental stage. Rhizosphere soil was collected using a sterile technique. Entire root systems were carefully excavated. After gently shaking off loosely attached bulk soil, roots with tightly adhering soil were placed in sterile phosphate-buffered saline (PBS, 0.1 M, pH 7.0) and vortexed for 5 min. The soil suspension was then centrifuged at 10,000 × g for 15 min at 4 °C. The pellet, designated as the rhizosphere soil fraction, was collected. Ten independent plants per group were sampled as biological replicates. All samples were immediately flash-frozen in liquid nitrogen and stored at −80 °C prior to DNA extraction.

### 2.2 DNA extraction and metagenomic sequencing

Total genomic DNA was extracted from 0.5 g of rhizosphere soil using a modified cetyltrimethylammonium bromide (CTAB) method (Doyle and Doyle, 1987). DNA concentration and purity were determined using a Qubit 3.0 Fluorometer (Invitrogen) and a NanoDrop spectrophotometer (Thermo Fisher Scientific), respectively. Integrity was verified by electrophoresis on a 1% agarose gel. Qualified DNA was used to construct paired-end (PE) libraries with an average insert size of 350 bp following the manufacturer’s protocol (Illumina). Library quality was assessed using an Agilent 2,100 Bioanalyzer. Metagenomic sequencing was performed on an Illumina HiSeq 2,500 platform (Baimaike Cloud, China) to generate 2 × 150 bp reads.

### 2.3 Metagenomic data processing and analysis

Raw sequencing reads were processed using Fastp (v0.23.0) (Chen et al., 2018) to remove low-quality bases (Q-score < 20), adapters, and reads shorter than 50 bp. Host-derived reads were identified and filtered by mapping clean reads to the tomato reference genome (assembly GCA_036512215.2) using bowtie2 (v2.5.4) (Langmead and Salzberg, 2012). *De novo* assembly of the high-quality, host-filtered reads was performed using MEGAHIT (v1.1.2) (Li et al., 2015) with default parameters. Open reading frames (ORFs) were predicted from assembled contigs (≥ 300 bp) using MetaGeneMark (v3.38) (Zhu et al., 2010). A non-redundant gene catalog was constructed by clustering predicted protein sequences at 90% amino acid identity and 80% coverage using MMseqs2 (v12-113e3) (Mirdita et al., 2021).

Representative sequences from the non-redundant catalog were taxonomically assigned by alignment against the NCBI non-redundant (NR) database using DIAMOND (Buchfink et al., 2015) with an e-value threshold of 1e^−5^. Functional potential was annotated by querying sequences against the Kyoto Encyclopedia of Genes and Genomes (KEGG, release 2023-01), Clusters of Orthologous Groups (COG), Gene Ontology (GO), eggNOG (v5.0), Pfam (v35.0), and SwissProt databases using DIAMOND. Specialized functional profiles were obtained by alignment against the Carbohydrate-Active Enzymes (CAZy) database and the Comprehensive Antibiotic Resistance Database (CARD) using hmmscan (HMMER v3.3.2) and the Resistance Gene Identifier (RGI v6.0.0), respectively. Differential abundance of taxonomic groups and functional genes between HRS and IRS was assessed using the two-tailed Wilcoxon rank-sum test with Benjamini–Hochberg false discovery rate (FDR) correction for multiple comparisons. For PERMANOVA, we used Bray–Curtis dissimilarity with 9,999 permutations, as implemented in the vegan package (v2.6–4) in R. Significance was defined as an *P*_FDR_ < 0.05.

### 2.4 Microbial co-occurrence network analysis

To ensure comparability between groups, all samples were rarefied to an equal sequencing depth of 40,000 reads per sample prior to network construction. Microbial co-occurrence networks were then constructed separately for HRS and IRS groups using the rarefied data. Spearman’s rank correlation coefficients were calculated between the relative abundances of microbial genera (with prevalence > 20% and relative abundance > 0.01%). Only robust correlations (|*ρ*| > 0.7) with statistical significance (*P*_FDR_ < 0.001) were retained to construct the network. Network visualization and topological properties (including node number, edge number, average degree, average path length, clustering coefficient, and modularity) were analyzed using the igraph package (v1.5.1) in R. Gephi (v0.10.1) was used for network visualization.

### 2.5 Functional redundancy calculation

Functional redundancy was defined as the number of taxa contributing to the same functional category, reflecting the community’s buffering capacity against functional loss (Louca et al., 2018). To quantify this, we calculated functional redundancy as the ratio of genus richness (number of genera) to functional richness (number of KEGG Orthology groups) for each sample. A higher ratio indicates greater functional redundancy, meaning that multiple taxa are capable of performing similar functions.

## 3 Results and analysis

### 3.1 General characteristics of metagenomic sequencing results

Following removal of low-quality reads and host-derived sequences, we obtained 42,357,844 high-quality reads from 20 metagenomic libraries, with individual samples containing between 40,052,644 and 49,678,908 reads. All samples exhibited exceptional base-call quality, with Q20 > 99% and Q30 > 98% (representing bases with phred quality scores > 20 and > 30, respectively). This suggests that the dataset was sufficient for subsequent statistical analysis (Supplementary Table S1). The metagenomic assembly exhibited comprehensive genomic coverage (255–487 Mbp), with GC content (57.51–62.59%) falling within the expected range for microbial communities and maximum contig lengths reaching 167.4 kbp, indicating adequate assembly quality for subsequent functional and taxonomic analyses, although the moderate N50 values (499–655 bp) suggest some degree of fragmentation in the assembled sequences (Supplementary Table S2). In addition, a total of 8,184,379 unigenes were identified from the 20 samples, meeting identity and coverage thresholds of over 90 and 80%, respectively. The unigene counts were 5,824,159 in the HRS group and 4,684,165 in the IRS group, with significantly fewer unigenes in the infected group (Wilcoxon rank-sum test, *p* < 0.001).

### 3.2 Root-knot nematode (RKN) infection drives structural and diversity shifts in rhizosphere microbiota

To systematically elucidate the overall impacts of RKN infection on the rhizosphere microbial community, we first compared the structural and diversity differences of microbial communities between RKN-infected plants (IRS) and from healthy plants (HRS).

**Figure 1:**
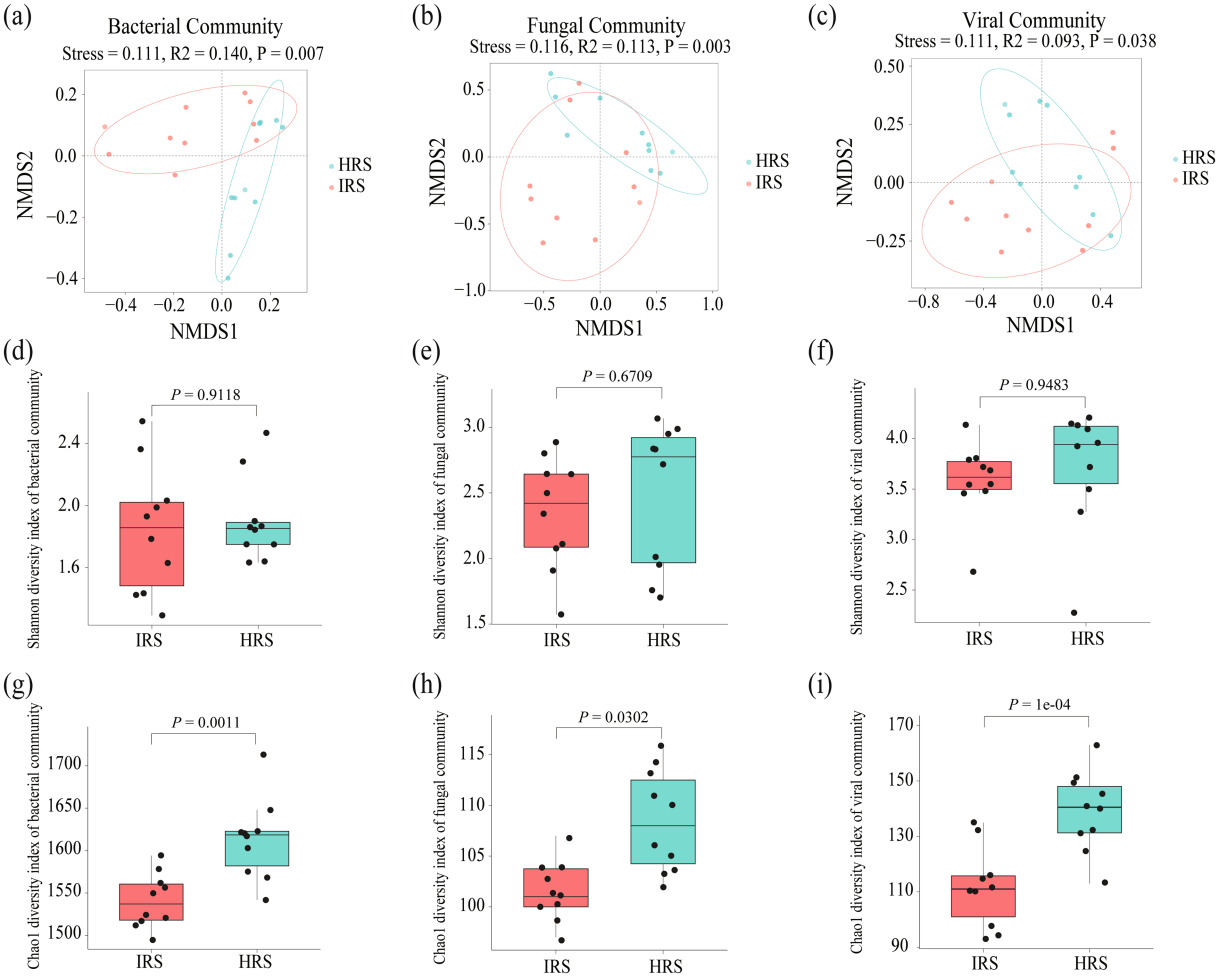
Structural and diversity shifts in rhizosphere microbiota driven by root-knot nematode (RKN) infection. **(a–c)** Non-metric multidimensional scaling (NMDS) ordination plots based on Bray–Curtis dissimilarity, showing the overall structural differences in bacterial **(a)**, fungal **(b)**, and viral **(c)** communities between healthy (HRS) and RKN-infected (IRS) rhizosphere soils. Each point represents a sample. Statistical significance was assessed by PERMANOVA (9,999 permutations, Bray–Curtis), with stress values and *R*^2^ indicated. **(d–f)** Boxplots showing the Shannon diversity index of bacterial **(d)**, fungal **(e)**, and viral **(f)** communities. No significant differences were observed between HRS and IRS (Wilcoxon rank-sum test, *p* > 0.05). **(g–i)** Boxplots showing the Chao1 richness index of bacterial **(g)**, fungal **(h)**, and viral **(i)** communities. Significant increases in richness were observed in HRS for all three microbial domains (Wilcoxon rank-sum test; *p* values as indicated). HRS, healthy rhizosphere soil; IRS, infected rhizosphere soil.

Non-metric multidimensional scaling (NMDS) ordination coupled with permutational multivariate analysis of variance (PERMANOVA) revealed a significant reshaping effect of RKN infection on the overall structure of rhizosphere microbial communities. At the genus level, distinct community structural differences between HRS and IRS were detected for bacteria (*R*^2^ = 0.140, *p* = 0.007), fungi (*R*^2^ = 0.113, *p* = 0.003), and viruses (*R*^2^ = 0.093, *p* = 0.038) (Figures 1a–c). In stark contrast, the archaeal community structure remained stable without significant shifts (*R*^2^ = 0.087, *p* = 0.161) (Supplementary Figure S1a). A detailed summary of taxonomic annotations across samples at each phylogenetic level is provided in Supplementary Table S3, and the inter-group comparative analysis of archaeal phyla between IRS and HRS is shown in Supplementary Table S4. These results confirmed that RKN infection is a key driver of structural changes in the rhizosphere bacterial, fungal, and viral communities.

Analysis of alpha diversity revealed more detailed patterns. The Chao1 richness index of bacterial, fungal, and viral communities was significantly elevated in HRS (bacteria: *p* = 0.0011; fungi: *p* = 0.0302; viruses: *p* < 0.001) (Figures 1g–i). In contrast, there was no significant difference in the Chao1 richness index of the archaeal community between HRS and IRS (archaea: *p* = 0.166). However, the Shannon diversity index, which integrates both richness and evenness, showed no significant differences between HRS and IRS across all microbial domains (archaea, bacteria, fungi, and viruses) (Supplementary Figure S1b, Figures 1d–f).

Taken together, these results suggested that RKN infection specifically regulates rhizosphere microbial richness. Although overall richness (Chao1) was significantly lower in IRS, we observed an increase in the relative abundance of certain low-abundance bacterial, fungal, and viral taxa in IRS, suggesting that nematode infestation may selectively enrich specific rare taxa while reducing overall diversity. No detectable effect on archaeal richness was observed. Importantly, the stability of Shannon diversity across all domains indicated that the infection selectively recruits and enriches a subset of rare taxa without destabilizing the indigenous dominant communities. This pattern reflected a targeted response of the rhizosphere microbiome to RKN. These findings lay a critical taxonomic foundation for further dissecting the functional implications of microbial community shifts under RKN.

### 3.3 Identification and ecological implications of differentially abundant microbial taxa in rhizosphere soils under nematode infection

To identify the specific microbial taxa driving the community structural shifts observed in Section 3.2, we performed differential abundance analysis (DAA) at both the phylum and genus levels across all three microbial domains (bacteria, fungi, and viruses).

**Figure 2:**
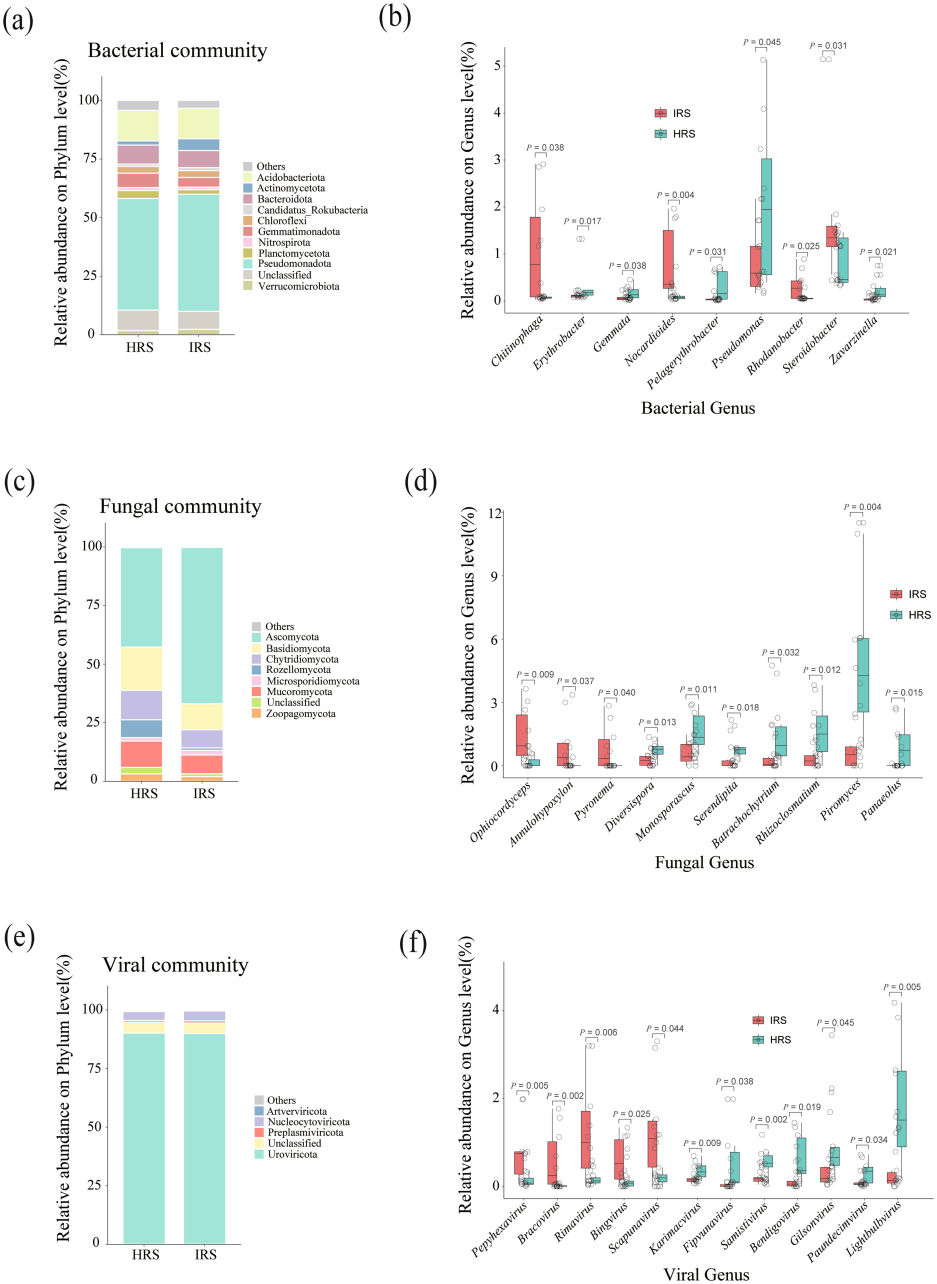
Differential abundance of rhizosphere microbial taxa in response to RKN infection. **(a,c,e)** Bar charts of dominant phyla in bacterial **(a)**, fungal **(c)**, and viral **(e)** communities in HRS (blue) and IRS (orange). Phyla with statistically significant differences (two-tailed Wilcoxon rank-sum test with Benjamini–Hochberg FDR correction, *P*_FDR_ < 0.05) between groups are indicated with asterisks and labeled. **(b,d,f)** Volcano plots of differentially abundant genera in bacterial **(b)**, fungal **(d)**, and viral **(f)** communities. Each point represents a genus. The *x*-axis indicates the log₂ fold change (IRS/HRS), and the y-axis shows the -log₁₀ of *P*_FDR_. Points are colored based on their enrichment pattern: red (significantly enriched in IRS), blue (significantly enriched in HRS), and gray (non-significant). Key genera discussed in the text are explicitly labeled. Key genera discussed in the text are explicitly labeled on the *x*-axis of the volcano plots **(b,d,f)**. HRS, healthy rhizosphere soil; IRS, infected rhizosphere soil; FDR, false discovery rate.

At the phylum level, the bacterial community exhibited a distinct taxonomic shift: the relative abundance of Pseudomonadota was significantly elevated in IRS compared to HRS (*P*_FDR_ = 0.034), whereas no statistically significant differences were detected among the remaining dominant bacterial phyla (Figure 2a, Supplementary Table S5). For the fungal community, Ascomycota (*P*_FDR_ = 0.021) and Microsporidia (*P*_FDR_ = 0.022) were significantly enriched in IRS, while Basidiomycota showed a higher relative abundance in HRS (*P*_FDR_ = 0.015) (Figure 2c, Supplementary Table S6). In the viral community, only the low-abundance phylum Phixviricota was differentially abundant between the two groups, with a higher enrichment in HRS (*P*_FDR_ = 0.048); no notable variations were found in other dominant viral phyla (Figure 2e, Supplementary Table S7).

To further pinpoint the key responsive taxa, we performed DAA on the top 50 most abundant genera within each microbial domain. The bacterial community displayed pronounced differential enrichment patterns with clear functional relevance (Figure 2b, Supplementary Table S8). IRS was selectively enriched with functionally specialized bacterial genera involved in critical soil biogeochemical processes: *Chitinophaga* (*P*_FDR_ = 0.038, effect size = −0.915), a genus well-documented for chitin degradation and associated with nematode-suppressive soils, consistent with the observed enrichment of chitin-degrading functions in IRS; *Nocardioides* (*P*_FDR_ = 0.004, effect size = −0.797) and *Steroidobacter* (*P*_FDR_ = 0.031, effect size = −0.662), which are associated with the decomposition of complex organic matter; and *Rhodanobacter* (*P*_FDR_ = 0.026, effect size = −0.790), implicated in nitrogen cycling and often enriched in stressed soils, aligning with the functional shifts toward stress response and altered nutrient metabolism. Notably, the opportunistic pathogenic genus *Pseudomonas* was also significantly enriched in IRS (*P*_FDR_ = 0.045, effect size = 0.696)—a finding that suggests a potential mechanistic link between nematode infection, pathogen proliferation, and rhizosphere dysbiosis. In sharp contrast, HRS was preferentially colonized by taxa typically adapted to marine or high-salinity environments, including *Pelagerythrobacter* (*P*_FDR_ = 0.031, effect size = 0.770) and *Erythrobacter* (*P*_FDR_ = 0.017, effect size = 0.519), suggesting a potential link between these taxa and the maintenance of a stable, healthy rhizosphere.

Differential shifts in the fungal community revealed a clear divergence in ecological functional guilds (Figure 2d, Supplementary Table S9). HRS was significantly enriched with taxa that form mutualistic associations with plants, including the arbuscular mycorrhizal fungus *Diversispora* (*P*_FDR_ = 0.013, effect size = 0.509), the beneficial endophyte *Serendipita* (*P*_FDR_ = 0.018, effect size = 0.685), and the saprotrophic fungus *Piromyces* (*P*_FDR_ = 0.004, effect size = 0.841)—a genus highly abundant in HRS that plays a key role in plant polysaccharide degradation. Additional taxa enriched in HRS included *Monosporascus* (*P*_FDR_ = 0.011, effect size = 0.577), *Batrachochytrium* (*P*_FDR_ = 0.032, effect size = 0.612), *Rhizoclosmatium* (*P*_FDR_ = 0.012, effect size = 0.771), and *Panaeolus* (*P*_FDR_ = 0.015, effect size = 0.922), further underscoring the critical role of plant-fungal symbiosis and specialized saprotrophy in maintaining rhizosphere health. Conversely, IRS harbored a higher abundance of taxa with pathogenic or saprotrophic traits: the insect pathogen *Ophiocordyceps* (*P*_FDR_ = 0.009, effect size = −0.740), along with the saprotrophic genera *Annulohypoxylon* (*P*_FDR_ = 0.037, effect size = −0.688) and *Pyronema* (*P*_FDR_ = 0.040, effect size = −0.666), which are capable of degrading recalcitrant complex polymers.

The viral community also underwent substantial taxonomic restructuring in response to nematode infection (Figure 2f, Supplementary Table S10). Relative to HRS, IRS was selectively enriched with several viral genera, including *Pepyhexavirus* (*P*_FDR_ = 0.005, effect size = −0.901), *Bracovirus* (*P*_FDR_ = 0.002, effect size = −0.892), *Rimavirus* (*P*_FDR_ = 0.006, effect size = −0.875), *Bingvirus* (*P*_FDR_ = 0.025), and *Scapunavirus* (*P*_FDR_ = 0.044, effect size = −0.813). In contrast, HRS was enriched for a distinct set of viral genera [e.g., *Lightbulbvirus* (*P*_FDR_ = 0.005, effect size = 0.900), *Samistivirus* (*P*_FDR_ = 0.002, effect size = 0.675), *Karimacvirus* (*P*_FDR_ = 0.009, effect size = 0.545), *Fipunavirus* (*P*_FDR_ = 0.038, effect size = 0.656), *Bendigovirus* (*P*_FDR_ = 0.019, effect size = 0.703), *Gilsonvirus* (*P*_FDR_ = 0.045, effect size = 0.732), and *Paundecimvirus* (*P*_FDR_ = 0.034, effect size = 0.845)]. Given the obligate parasitic nature of most of these viruses, which primarily infect bacterial hosts, the enrichment of specific viral genera in IRS likely reflects a concomitant increase in the activity or abundance of their respective bacterial hosts under nematode-induced disease stress.

In summary, root-knot nematode infection not only drives substantial structural rearrangements in rhizosphere bacterial, fungal, and viral communities but also mediates the selective enrichment of distinct functional guilds in HRS and IRS. Specifically, IRS is characterized by the proliferation of opportunistic pathogens (e.g., *Pseudomonas*), taxa involved in organic matter decomposition and nutrient cycling (e.g., *Chitinophaga*, *Rhodanobacter*), and host-associated viruses (e.g., *Bracovirus*, *Pepyhexavirus*). In contrast, HRS is dominated by beneficial plant-associated symbionts (e.g., *Diversispora*, *Serendipita*), specialized saprotrophs (e.g., *Piromyces*), and halophilic/marine-related bacterial taxa (e.g., *Pelagerythrobacter*, *Erythrobacter*).

### 3.4 Divergent responses of microbial interaction networks to RKN

**Figure 3:**
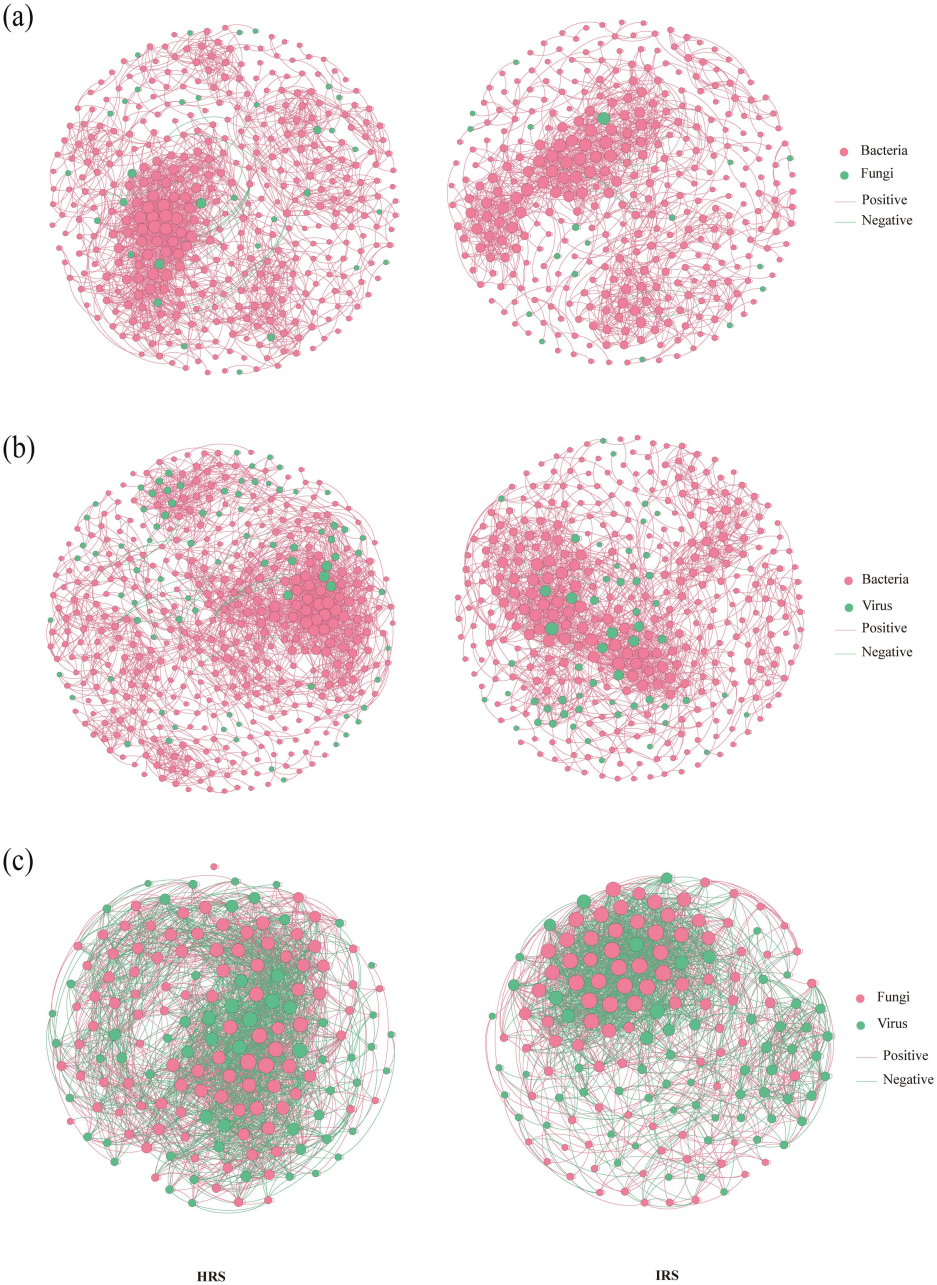
Divergent responses of intra-kingdom microbial co-occurrence networks to RKN infection. **(a–c)** Intra-kingdom networks: bacteria-bacteria **(a)**, fungi-fungi **(b)**, and virus-virus **(c)** association in HRS (left) and IRS (right). Co-occurrence networks were constructed using samples rarefied to an equal sequencing depth (40,000 reads per sample). Nodes represent microbial taxa, and edges represent significant Spearman correlations (|*ρ*| > 0.7) with *P*_FDR_ < 0.001. Edge colors denote positive (green) and negative (red) correlations. HRS, healthy rhizosphere soil; IRS, infected rhizosphere soil; FDR, false discovery rate.

**Figure 4:**
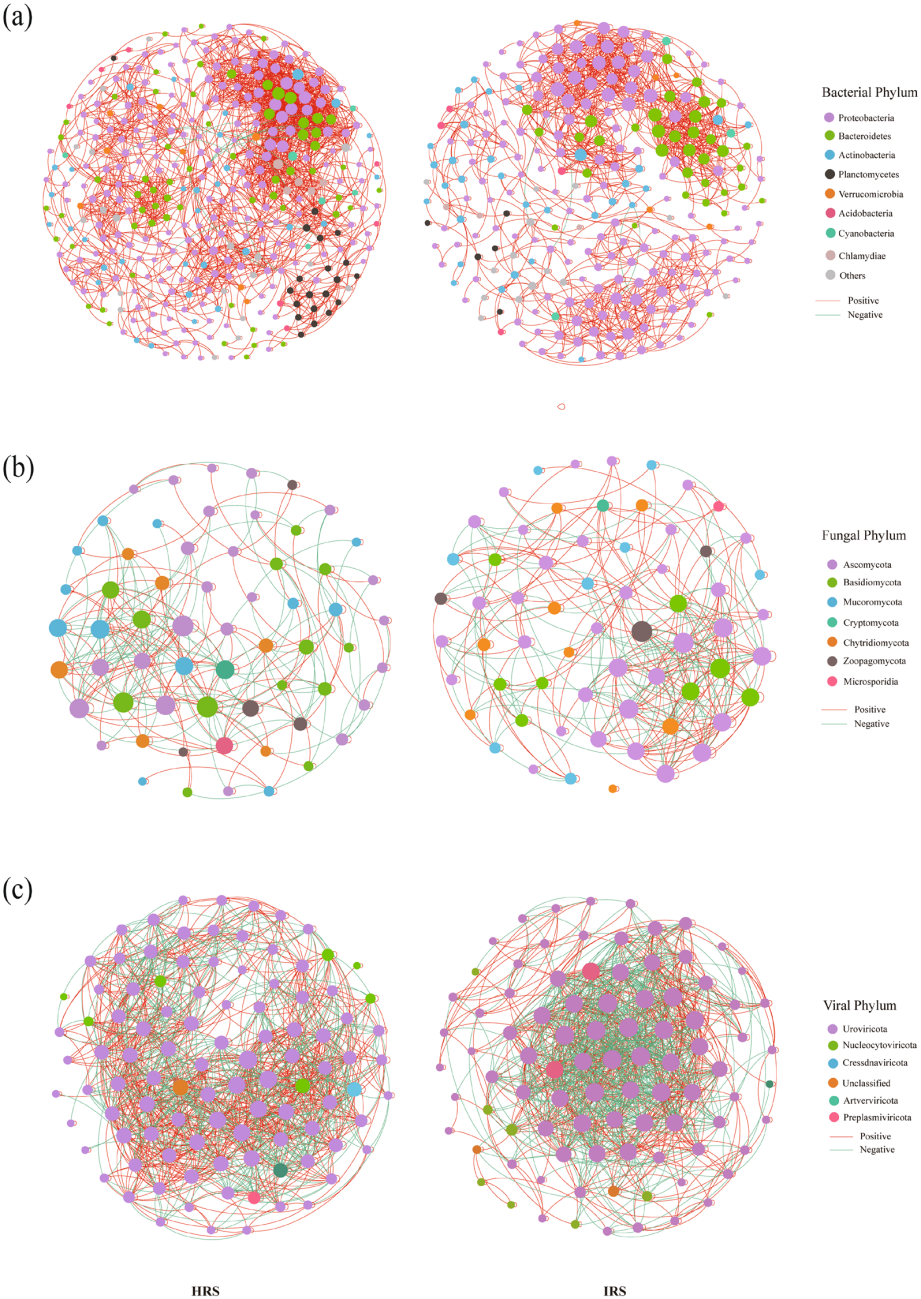
Divergent responses of inter-kingdom microbial co-occurrence networks to RKN infection. **(a–c)** Inter-kingdom networks: bacteria-fungi **(a)**, bacteria-virus **(b)**, and fungi-virus **(c)** association in HRS (left) and IRS (right). Co-occurrence networks were constructed using samples rarefied to an equal sequencing depth (40,000 reads per sample). Nodes represent microbial taxa, and edges represent significant Spearman correlations (|ρ| > 0.7) with *P*_FDR_ < 0.001. Edge colors denote positive (green) and negative (red) correlations. HRS, healthy rhizosphere soil; IRS, infected rhizosphere soil; FDR, false discovery rate.

To explore how rhizosphere microbial interactions respond to RKN stress, we constructed and compared intra-kingdom (bacteria–bacteria, fungi–fungi, virus–virus) (Figure 3) and inter-kingdom (bacteria–fungi, bacteria–virus, fungi–virus) co-occurrence networks between HRS and IRS (Figure 4). All networks were constructed using samples rarefied to an equal sequencing depth to ensure comparability between groups (see Methods). Therefore, the observed differences in network topology between HRS and IRS reflect genuine biological shifts rather than artifacts of sequencing depth.

Intra-kingdom network analysis revealed divergent response strategies among bacterial, fungal, and viral communities (Figure 3, Supplementary Table S11). The bacterial–bacterial network in IRS exhibited a marked reduction in scale, with nodes decreasing from 375 to 255, edges from 2,376 to 1,390, and average degree from 12.672 to 10.902 (Figure 3a). Although positive correlations remained dominant (> 99%), the contraction of network size indicates that RKN significantly weakened widespread bacterial interactions, potentially reducing community structural stability and functional redundancy. In contrast, the fungal–fungal network in IRS showed stronger internal cohesion: node numbers remained comparable (63 vs. 61), but edges increased from 280 to 344, average degree from 4.607 to 5.952, and the proportion of positive edges rose from 58.57 to 64.83% (Figure 3b). This suggested that fungal communities may enhance internal positive cooperation as a compensatory stress response, forming a more tightly connected subcommunity. The viral–viral network displayed a distinct pattern: nodes (91 vs. 101) and edges (1,069 vs. 1,234) slightly decreased, but average degree increased from 14.98 to 16.011, indicating tighter connections between viral nodes under stress. Most notably, the proportion of positive edges dropped significantly from 63.94 to 55.00% (Figure 3c), implying a substantial increase in antagonistic or competitive interactions within the viral community—likely reflecting intensified resource competition for bacterial or fungal hosts.

Inter-kingdom analysis further revealed a community-wide attenuation of interactions (Figure 4, Supplementary Table S11). Both bacteria–fungi and bacteria–virus networks in IRS showed reductions in nodes, edges, and average degree. However, positive correlations remained extremely high (> 99.5%) (Figures 4a,b), suggesting that the preserved cross-kingdom interactions are predominantly conserved mutualistic or symbiotic associations. The fungi–virus network also exhibited reduced scale and connectivity (average degree: 13.597 vs. 17.148), with the proportion of positive edges decreasing from 59.41 to 54.78% (Figure 3c). This shift indicated a relative increase in antagonistic or competitive interplay between fungi and viruses, potentially reflecting enhanced resource competition or viral lytic activity against fungal hosts.

Collectively, our findings suggested that the overall interaction stability of the microbial community is systematically reshaped under disease stress. The significant contraction of bacterial networks directly reduced their topological robustness. While fungal networks showed enhanced internal cohesion, their key cross-kingdom interactions with bacteria and viruses were generally weakened, potentially leading to abnormally high functional modularity and impaired collaboration between functional modules. Meanwhile, viral networks maintain high connectivity but exhibit a marked increase in internal competition, which might further exacerbate community dynamic instability. This combined pattern—bacterial interaction collapse, fungal internal tightening, intensified viral competition, and attenuated cross-kingdom interactions—points to reduced functional coordination and ecosystem resilience in the infected rhizosphere, which is typically linked to increased functional vulnerability under prolonged stress.

### 3.5 RKN-induced reprogramming of microbial functional potential in the rhizosphere

**Figure 5:**
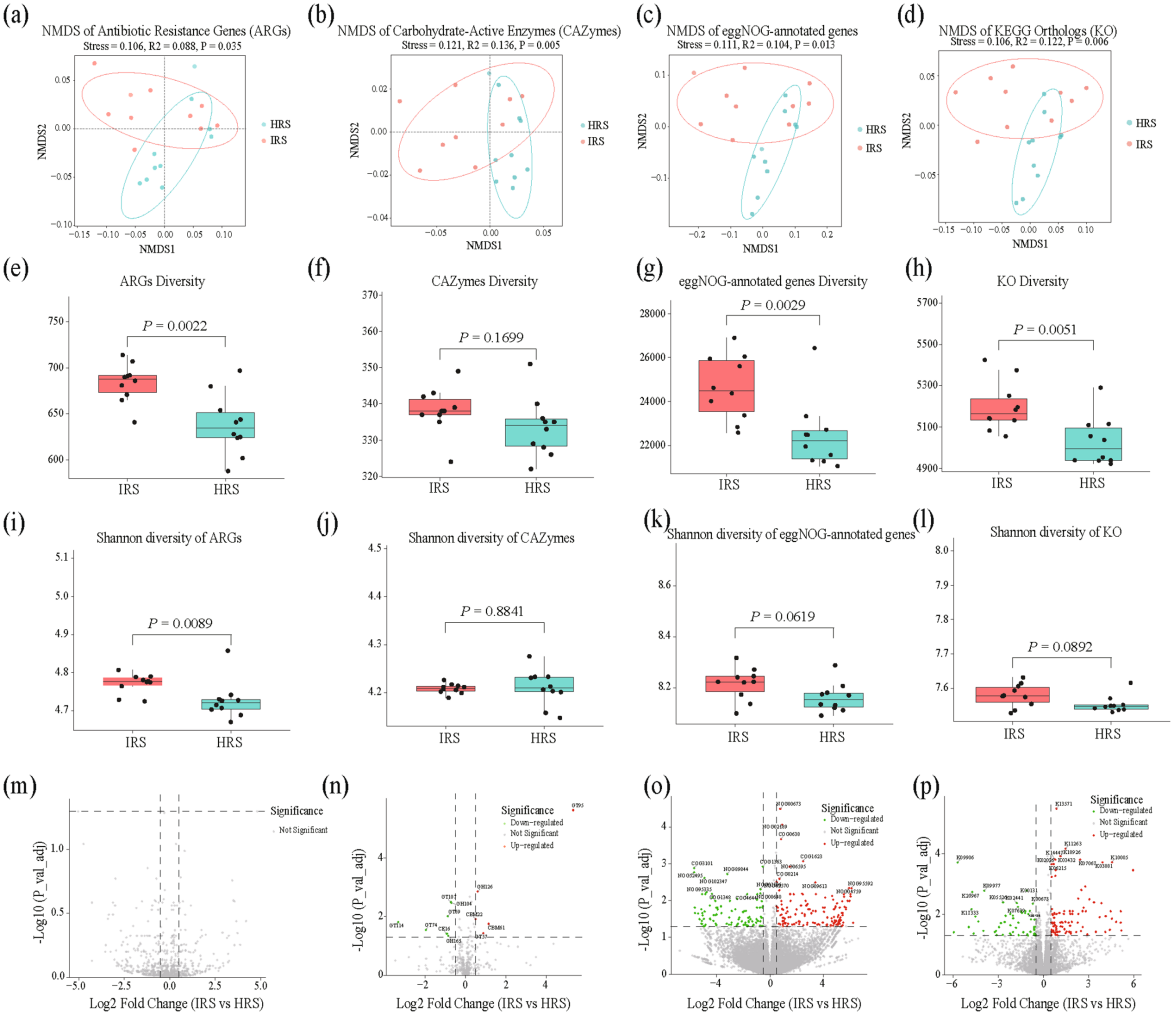
Stress-induced reprogramming of microbial functional potential in the rhizosphere microbiome under RKN infection. **(a–d)**: NMDS ordination (Bray–Curtis) of ARGs **(a)**, CAZymes **(b)**, eggnog **(c)**, and KO terms **(d)** in HRS and IRS. Statistical significance was assessed by PERMANOVA (9,999 permutations, Bray–Curtis), with stress values and *R*^2^ indicated. **(e–l)** Shannon diversity of ARGs **(e,i)**, CAZymes **(f,j)**, eggNOG **(g,k)**, and KO terms **(h,l)** between HRS and IRS. Asterisks denote significant differences (two-tailed Wilcoxon rank-sum test with Benjamini–Hochberg FDR correction, *p* < 0.05). **(m–p)** Heatmaps displaying the relative abundance (*Z*-score normalized) of significantly differentially abundant functional features between HRS and IRS for ARGs **(m)**, CAZymes **(n)**, eggNOG **(o)**, and KO terms **(p)**. Rows represent individual genes or orthologs, and columns represent sample groups. Functional categories are annotated on the right. HRS, healthy rhizosphere soil; IRS, infected rhizosphere soil; ARGs, antibiotic resistance genes; CAZymes, carbohydrate-active enzymes; KO, KEGG Orthology; NMDS, non-metric multidimensional scaling; PERMANOVA, permutational multivariate analysis of variance; FDR, false discovery rate.

To dissect the response of rhizosphere microbial functional potential to root-knot nematode infection, we systematically compared the functional differences between HRS and IRS based on metagenomic data, including antibiotic resistance genes (ARGs), carbohydrate-active enzymes (CAZymes), eggNOG orthologous groups, and KEGG Orthology (KO) terms (Figures 5a–p; Supplementary Tables S12–S15).

NMDS ordination revealed a clear separation in the overall microbial functional profiles between HRS and IRS across all analyzed functional categories (Figures 5a–d). Specifically, significant differences were observed for ARGs (stress = 0.106, *R*^2^ = 0.088, *p* = 0.035), CAZymes (stress = 0.121, *R*^2^ = 0.136, *p* = 0.005), eggNOG-annotated genes (stress = 0.111, *R*^2^ = 0.104, *p* = 0.013), and KO terms (stress = 0.106, *R*^2^ = 0.122, *p* = 0.006), which confirmed that nematode infection comprehensively reshapes the functional composition of the rhizosphere microbial community.

Differential functional analysis uncovered a fundamental shift in metabolic focus (Figures 5m–p). In IRS, functional genes associated with core energy production, stress response, and protein turnover were significantly upregulated (Supplementary Table S13). These included key components of the respiratory chain (e.g., NADH: ubiquinone oxidoreductase chains 4 and 6, K03881 and K03884; cytochrome c oxidase subunits 1 and 2, K02256 and K02261) and proteasome *α*/*β* subunits (K03432, K03433). Conversely, genes involved in photosynthesis (e.g., photosystem I P700 chlorophyll an apoprotein A1, K02689; photosystem II CP47 chlorophyll apoprotein, K02704), chlorophyll synthesis (e.g., magnesium-protoporphyrin IX monomethyl ester cyclase, K04035), and partial amino acid transport were generally downregulated (Supplementary Table S14).

In terms of carbohydrate metabolism, IRS exhibited specific adjustments in degradation pathways for target substrates. Analysis at the CAZyme family level showed that genes belonging to the CBM22 family (associated with xylan binding) and the GH126 family (with α-amylase activity) were significantly enriched in IRS. In contrast, genes encoding chitin/peptidoglycan acetylesterase (CE16), peptidoglycan lytic transglycosylase (GH104), and multiple glycosyltransferase families (e.g., GT69, GT114) were significantly downregulated (Supplementary Table S12). Although there was no significant difference in the overall Shannon diversity of CAZymes between the two groups (*p* = 0.884), the total diversity of eggNOG and KO functions was significantly reduced in IRS (Figures 5i–l). This pattern indicated that under nematode stress, the microbial community contracts its broad metabolic network while retaining the capacity to degrade specific carbohydrates, redirecting resources toward core energy metabolism.

Notably, the ARG pool displayed a unique dynamic. The Shannon diversity of ARGs was significantly higher in IRS than in HRS (*p* = 0.0089) (Figure 5i). However, differential functional analysis (Figure 5m) showed that the abundance of the vast majority of individual ARGs did not change significantly. This pattern suggested that the expansion of the resistance gene pool under stress may result from low-level increases in numerous ARGs or accumulation via horizontal gene transfer, rather than the explosive amplification of a few genes (Supplementary Table S15). This could represent a strategy for the microbial community to enhance environmental adaptability under nematode-induced stress.

Functional redundancy, calculated as the ratio of genus richness to KO richness, was significantly lower in IRS compared to HRS (Wilcoxon rank-sum test, *p* = 0.047) (Supplementary Table S17). This indicates that under nematode stress, fewer microbial taxa are available to perform each functional category, reflecting a loss of functional redundancy.

### 3.6 Integrated co-functional network analysis unveils a streamlined Core architecture under disease stress

**Figure 6:**
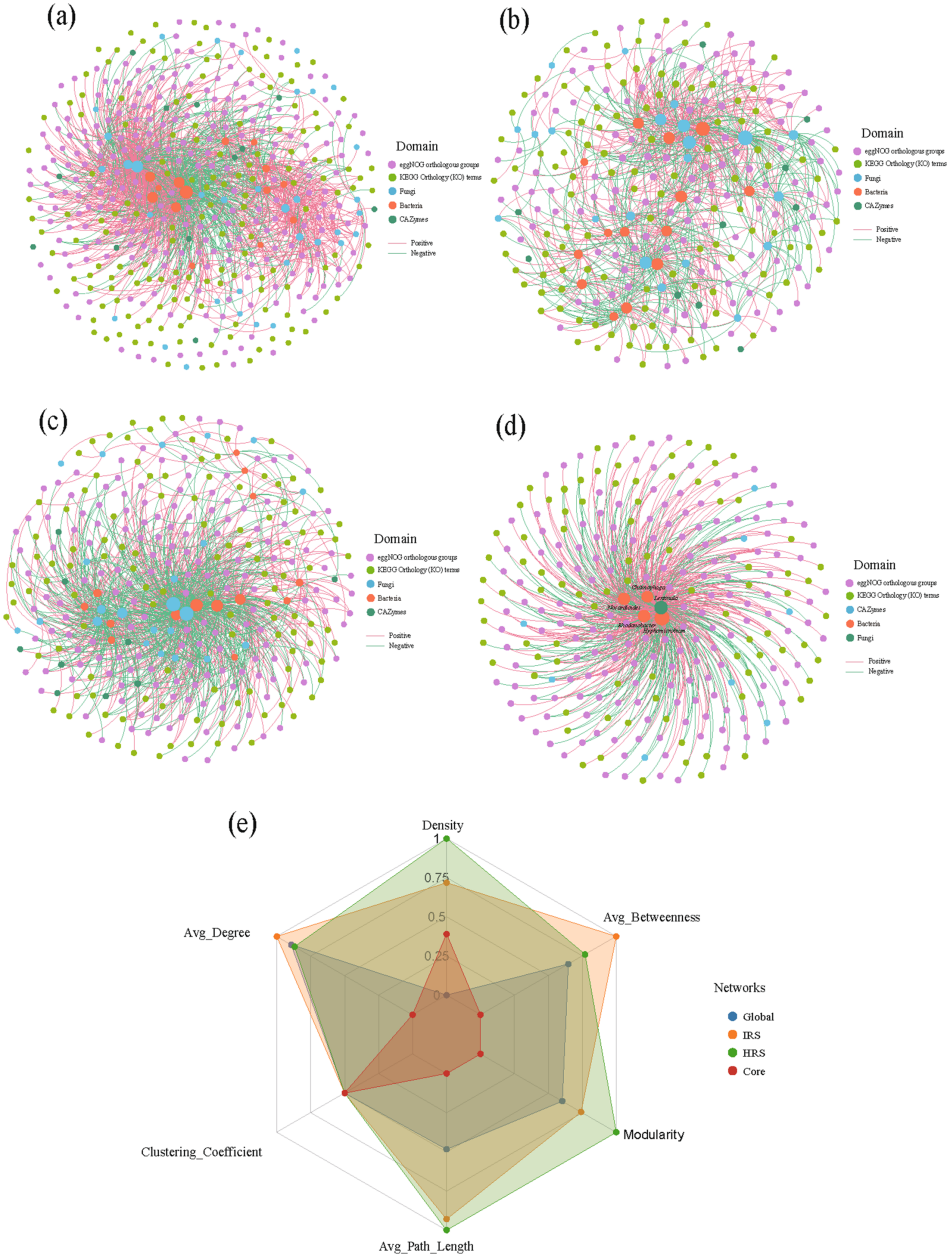
Integrated co-functional network analysis reveals a streamlined, centralized architecture under RKN stress. **(a)** Visualization of a global microbial-functional co-occurrence network, illustrating the integrated interactions between microbial taxa and functional genes. Networks were constructed using samples rarefied to an equal sequencing depth (40,000 reads per sample). Edges represent significant Spearman correlations (|ρ| > 0.7) with *P*_FDR_ < 0.001. Node types and colors: Circles represent microbial taxa (orange, bacteria; blue, fungi). Squares represent functional annotation units, colored by database: KEGG Orthology (KO) terms (green), eggNOG orthologous groups (yellow), and Carbohydrate-Active Enzymes (CAZymes, purple). Edge colors: Green lines indicate significant positive co-occurrence, red lines indicate negative co-occurrence. **(b,c)** Separate global co-functional networks for the healthy (HRS, **b**) and infected (IRS, **c**) rhizosphere microbiomes. The IRS network **(c)** exhibits an expanded number of nodes but a reduction in overall network density and modularity compared to the HRS network **(b)**. **(d)** The core response subnetwork extracted from the IRS global network, comprising the top 20% of nodes with the highest betweenness centrality. This streamlined architecture is functionally concentrated, dominated by gene nodes interconnected by a few keystone microbial hubs (labeled: *Chitinophaga*, *Hyphomicrobium*, *Nocardioides*, *Rhodanobacter*, *Lentinula*). **(e)** Radar chart comparing key topological properties across the three networks: HRS global (blue), IRS global (orange), and the IRS core subnetwork (green). Metrics include Density, Average Degree (Avg_Degree), Clustering Coefficient, Average Path Length (Avg_Path_Length), Modularity, and Average Betweenness Centrality (Avg_Betweenness). HRS, healthy rhizosphere soil; IRS, infected rhizosphere soil; KO, KEGG Orthology; CAZymes, carbohydrate-active enzymes; FDR, false discovery rate.

To systematically decipher the synergistic reorganization of microbial taxa and functional genes in response to disease stress, integrated co-functional networks were constructed for HRS and IRS. Comparative analyses of multi-dimensional topological properties were subsequently performed to unravel stress-induced structural dynamics (Figures 6a–e; Supplementary Table S16).

Microbial-functional co-occurrence networks analysis in HRS and IRS revealed that disease stress triggered substantial topological reorganization of the rhizosphere microbe-functional gene interaction network. The HRS-specific network exhibited characteristics of high density and strong modularity, with microbial nodes (14 bacteria and 21 fungi, accounting for 12.7% of total nodes) deeply integrated and tightly associated with functional annotation nodes (241 in total) (Figure 6b; Supplementary Table S13). This observation indicated that under steady-state conditions, the rhizosphere microbiome functions as a robust system with high functional redundancy and intensive internal interactions, capable of maintaining the stability of the rhizosphere microecosystem through a distributed network structure. In striking contrast, the IRS-specific network displayed an expanded node scale (321 vs. 276 nodes in HRS) with a notable increase in functional annotation nodes (284 vs. 241). Despite a reduction in overall network density, the average degree of the IRS network was slightly elevated (7.21 vs. 6.94 in HRS) (Figure 6C; Supplementary Table S16). Moreover, the IRS network exhibited a distinct centralized trend, with a marginal decrease in the proportion of microbial nodes to 11.5% (14 bacteria and 23 fungi). This topological shift intuitively reflected the adaptive transition of interaction patterns from distributed synergy under steady-state conditions to centralized emergency response under disease stress.

To precisely identify the core scaffold supporting network functionality, a core subnetwork was constructed by extracting the top 20% of nodes with the highest centrality from the global network (Figure 6D). This core network showed a strong bias toward functional genes, which accounted for 98.1% of total nodes (263 out of 268), with only 5 key microbial taxa (4 bacterial and 1 fungal taxa) serving as core connection hubs (Supplementary Table S16). These hub taxa were closely associated with a suite of core metabolic functional genes, primarily annotated in the KEGG Orthology (KO) terms and eggNOG orthologous groups databases. Notably, the bacterial and fungal hub taxa in the core subnetwork are *Chitinophaga*, *Hyphomicrobium*, *Nocardioides*, *Rhodanobacter*, and *Lentinula* which correspond to the differentially abundant microbial taxa identified in Section 3.3.

Comprehensive comparison of topological properties via radar plot (Figure 6E) further validated the aforementioned structural transitions. The IRS-specific network outperformed the HRS network in terms of average degree (7.21) and clustering coefficient, indicating the formation of tightly connected local hub clusters within the network under disease stress, which facilitates the rapid synergistic response of core functions. In contrast, the core subnetwork exhibited the highest betweenness centrality and average degree (7.5), fully confirming its pivotal bridging role in connecting different functional modules of the network and mediating key metabolic pathways (Supplementary Table S16).

Collectively, the quantitative analysis of network topological indices suggested that disease stress induces significant reorganization of microbe-functional gene interactions in the rhizosphere. By selecting core microbial hubs and key metabolic functional genes, the network forms a streamlined, functionally concentrated, and efficiently responsive yet potentially less stable core response architecture. This adaptive transition provides critical support for the microbiome to maintain the supply of core functions under stressful environments.

## 4 Discussion

### 4.1 RKN infection drives the disintegration of rhizosphere microbial networks and community structure

The stability of rhizosphere microbiome networks is critical for plant health and disease suppression capacity (Berendsen et al., 2012; Mendes et al., 2011). Our findings study suggests that RKN infection is a pivotal driver of rhizosphere microbial restructuring. This selective perturbation indicates that RKN infection exerts a differential filtering pressure on specific microbial taxa, likely mediated through changes in root exudate chemistry or the rhizosphere environment. This shift signifies a functional transition from a mutualism-dominated state to one geared toward saprotrophy and pathotrophy. This transition aligns with classical models describing microbiome dysbiosis and functional instability upon pathogen invasion (Lehmann et al., 2020; Schlaeppi and Bulgarelli, 2015).

Among the taxa significantly depleted in IRS, several are well-documented for their roles in disease suppression. The arbuscular mycorrhizal fungus *Diversispora* and the beneficial endophyte *Serendipita*, both significantly enriched in HRS (Figure 2d), are known to promote plant health and suppress soil-borne diseases by enhancing nutrient uptake and inducing systemic resistance (Berendsen et al., 2012). The saprotrophic fungus *Piromyces*, also enriched in HRS, contributes to plant polysaccharide degradation and may indirectly support plant defense through nutrient cycling. Conversely, *Pseudomonas*—a genus associated with both plant growth promotion and opportunistic pathogenicity—was significantly enriched in IRS (Figure 2B), which may reflect a shift toward a more pathogenic or stress-tolerant microbial community under nematode infestation. Although *Bacillus* and *Pasteuria* were not among the top differentially abundant genera in our dataset, their reduced relative abundance in IRS (Supplementary Table S8) is consistent with the hypothesized loss of suppressiveness. These findings suggest that the putative decline in rhizosphere disease suppression may result not only from the loss of specific beneficial taxa but also from the enrichment of potentially pathogenic or competitive microorganisms.

A key finding of this work is that RKN infection is associated with a systematic simplification and loss of modularity in microbial interaction networks, which may help explain the putative decline in rhizosphere suppressiveness. This provides a network-level mechanistic framework for understanding disease onset, suggesting how environmental stress erodes microbial network stability and function (Hernandez et al., 2021). We observed a significant reduction in the scale (node number, connectivity) and link density of intra-domain bacterial networks in IRS (Figure 4A). This erosion of network complexity and connectivity implies diminished functional redundancy and ecological resilience, a hallmark of stressed and functionally impaired soil ecosystems (Delgado-Baquerizo et al., 2016; Wagg et al., 2019). Intriguingly, intra-domain fungal networks displayed a contrasting strategy, with strengthened internal connectivity under stress (Figure 4B). This enhanced modular cohesion may represent an adaptive fungal strategy to preserve core functionality through intensified within-group cooperation. However, this within-domain consolidation occurs at the expense of cross-domain interactions. Critical inter-kingdom networks (e.g., bacteria-fungi, bacteria-viruses) in IRS showed severely attenuated scale and connectivity (Figures 3A-c). The resulting “tight within domains, loose between domains” topology exacerbates community modularization, potentially decoupling the ecological synergy between different functional groups and fragmenting the integrated micro-ecological niche. Since rhizosphere disease suppression relies on cross-kingdom synergies—where bacteria may produce antimicrobials, fungi provide spatial niches, and viruses modulate host dynamics—the structural disintegration of these interaction networks inevitably disables this collaborative mechanism, a principle central to the concept of microbiome-mediated plant protection (Berendsen et al., 2012). The specific collapse of bacteria-fungi interaction networks has been directly linked to loss of disease suppression (Tan et al., 2022), thereby crippling the community’s overall suppressiveness.

In conclusion, the impact of RKN infection transcends mere taxonomic turnover, effecting a holistic disruption of the rhizosphere’s interaction network. Our findings suggest that the putative collapse of disease-suppressive function may be rooted in the systemic failure of microbial cooperative networks. This underscores a paradigm shift: understanding rhizosphere health requires a focus on the dynamic web of interactions, not just a static catalog of species—a perspective central to modern microbiome ecology (Faust and Raes, 2012; Van Der Heijden and Hartmann, 2016). Ultimately, we emphasize that the maintenance of ecosystem function hinges on the stability of microbial relationships, not simply the presence of specific taxa, thereby aligning with the fundamental ecological principle that network architecture and resilience govern ecosystem outputs (Hernandez et al., 2021; Wagg et al., 2019).

Limitations and future directions. It is important to acknowledge that our study design—comparing the rhizosphere microbiomes of healthy versus gall-symptomatic plants—does not directly measure disease-suppressive function. A soil suppressiveness bioassay would be required to confirm whether the observed microbial changes translate to functional loss of suppression. Therefore, our conclusions should be interpreted as correlational and hypothesis-generating rather than definitive. Additionally, it is important to consider whether the observed microbial shifts are driven directly by the nematode or indirectly through nematode-induced changes in root exudates. Root exudates—including organic acids, amino acids, and secondary metabolites such as flavonoids—are primary drivers of rhizosphere microbiome assembly. While our data cannot definitively distinguish between these effects, it is plausible that RKN infestation induces changes in root exudation patterns, which in turn shape the rhizosphere microbiome. Furthermore, correlation-based co-occurrence networks may be influenced by underlying biomass differences and threshold selection. While we used rarefied data to minimize artifacts due to sequencing depth, future studies employing experimental validation (e.g., co-culture assays) or alternative approaches would be valuable to confirm these network-level patterns. Future studies integrating root exudate metabolomics, viral metatranscriptomics, and host strain-level resolution will be needed to disentangle these complex mechanisms.

### 4.2 Functional reprogramming and ecological adaptation of the microbiome under disease stress

Pathogen stress induces directional changes in the metabolic functions of microbial communities (Lai et al., 2025; Zheng et al., 2026), but the driving mechanisms remain divided (Korenblum et al., 2022; Lai et al., 2025).

Our metagenomic data provide direct evidence to address this controversy. We found that RKN infection triggered a significant restructuring of the functional potential in the rhizosphere microbiome, manifested as a directional shift in the overall functional centroid (Figure 5), aligning with known patterns of microbiome functional reorganization under biotic stress (Trivedi et al., 2020). Further analysis revealed significant enrichment of functional modules related to core energy metabolism (e.g., genes encoding respiratory chain complexes) and protein turnover (e.g., genes encoding proteasome subunits) in the infected rhizosphere soil, a stress-responsive metabolic shift documented in soil microbial communities (Jansson and Hofmockel, 2018). Concurrently, the abundance of genes associated with photosynthesis and certain biosynthetic pathways was markedly reduced. This shift in the functional profile reveals a clear transition in metabolic strategy: the microbial community reallocated resources from growth and synthesis towards the maintenance of basic survival, stress response, and rapid energy acquisition. This pattern of contraction towards core survival metabolism represents a common adaptive strategy of microbial communities facing adversity stress, as conceptualized in frameworks of microbial resource allocation under perturbation (Malik et al., 2020).

Through integrated analysis, we linked these functional changes to the dynamics of specific microbial taxa. Bacterial genera significantly enriched in the infected rhizosphere—such as *Chitinophaga* (chitin degradation), *Nocardioides* (organic matter decomposition), and *Rhodanobacter* (nitrogen cycling)—possess functional traits that align well with the observed enhancement in organic matter degradation and nutrient mineralization potential at the community level (Figure 2B), supporting the paradigm that shifts in microbial community composition drive corresponding changes in ecosystem functioning (Delgado-Baquerizo et al., 2016; Fierer et al., 2013). Crucially, co-occurrence network analysis integrating microbial taxa and functional gene data revealed that these differentially enriched taxa (e.g., *Chitinophaga*, *Hyphomicrobium*) acted as keystone hubs within the stress-perturbed network, exhibiting direct and dense connections with numerous core stress-response functional genes (Figure 6). This indicates that the functional reprogramming of the rhizosphere microbiome is not a process involving all members equally, but rather is driven and dominated by a subset of key taxa possessing specific metabolic capabilities, a phenomenon consistent with the concept of “microbial keystone taxa” governing microbiome structure and function (Banerjee et al., 2018; Zheng et al., 2021).

These findings suggest that nematode-induced functional remodeling of the rhizosphere microbiome represents a non-selective metabolic load driven by combined host immune and pathological stress. The physical damage and persistent physiological stress inflicted on roots by nematodes likely alter the flux and composition of root exudates, thereby non-specifically enriching copiotrophic microorganisms adept at utilizing simple carbon sources and labile organic compounds (Chaparro et al., 2014; Fierer et al., 2007). This process drives a systemic upregulation of functions related to rapid energy acquisition and organic matter mineralization, which aligns more closely with a fundamental metabolic restructuring than with a specific adaptation to the nematode’s lifecycle (Manzoni et al., 2012; Trivedi et al., 2020). Although certain upregulated functions (e.g., chitin degradation) could theoretically be indirectly linked to the parasitism process, our data did not support a specific association with RKN infection. Furthermore, the reduction in beneficial symbiotic taxa (e.g., *Diversispora*) and the significant decline in functional gene diversity, together with the reduction in functional redundancy (Supplementary Table S17), suggests that this adaptation may come at the cost of the system’s long-term ecological stability (Delgado-Baquerizo et al., 2016; Wagg et al., 2019).

In summary, this study suggests that the infection-induced contraction of microbial functions is less a specific response to the nematode and more a survival adaptation of the system under duress, reflecting a general stress-response strategy in microbial communities (Trivedi et al., 2020). While this adaptation may sustain basic functions in the short term, it does so by sacrificing long-term resilience and multifunctionality, demonstrating a critical trade-off between immediate survival and sustained ecosystem functioning—a principle central to ecological theory (Delgado-Baquerizo et al., 2016; Tilman, 1999). This perspective opens a new window for understanding the complex interactions among plants, microbes, and pathogens through the lens of ecological trade-offs, aligning with calls for a more integrative, function-focused framework in plant-microbiome research (Bardgett and Van Der Putten, 2014).

### 4.3 Viral-host dynamics and potential lysogenic induction under nematode stress

The inclusion of viral metagenomics in this study provides a unique opportunity to explore viral-host dynamics in the context of RKN infestation. The significant differences in viral genera such as *Samistivirus* and *Lightbulbvirus* between HRS and IRS are particularly intriguing. Several viral genera enriched in IRS (e.g., *Bracovirus*, *Pepyhexavirus*) are known to associate with bacterial hosts, and their increased abundance may reflect shifts in host bacterial populations. More importantly, the observed enrichment of lysogenic-associated viral genera raises the possibility that environmental stress induced by nematode infestation may trigger prophage induction, shifting viral communities from lysogenic to lytic cycles. Such a shift could contribute to bacterial cell lysis and thereby destabilize the bacterial co-occurrence network, potentially exacerbating the “collapse” of bacterial interactions observed in IRS. Although direct evidence of phage induction requires metatranscriptomic validation, this hypothesis is consistent with the concurrent reduction in bacterial network complexity and the increase in viral competition (as indicated by the reduced proportion of positive edges in viral-viral networks). Future studies integrating viral metatranscriptomics and host strain-level resolution will be needed to test this mechanistic model.

## 5 Conclusion

In conclusion, this study suggests that RKN infection is associated with a fundamental ecological shift in the rhizosphere microbiome—from a complex, cooperative, and functionally diverse network to a simplified, survival-oriented state. This shift is characterized by a decline in microbial diversity, a collapse of cross-domain interaction networks, and a contraction of metabolic functions toward core energy acquisition and stress response. While indicative of an acute adaptive response, this reconfiguration comes at the cost of reduced ecological stability and functional redundancy, which may help explain the putative loss of disease-suppressive capacity in the rhizosphere.

These findings provide a network-level mechanistic understanding of plant–microbe–pathogen interactions, highlighting that dysbiosis under pathogen stress is not merely a compositional change but an ecosystem-wide trade-off between immediate survival and long-term resilience. From an applied perspective, our results suggest that effective microbiome-based management of soil-borne diseases should focus on restoring or preserving the structural and functional complexity of rhizosphere microbial networks, rather than relying solely on single antagonistic agents. Future interventions could target the identification and modulation of keystone taxa and functional modules to enhance the innate stability and suppressiveness of agricultural soil ecosystems.

## Data Availability

The datasets presented in this study can be found in online repositories. The names of the repository/repositories and accession number(s) can be found at: https://www.ncbi.nlm.nih.gov/, PRJNA1414134.

## Supplementary material

Supplementary Figure S1 Structural and compositional stability of the archaeal community in response to RKN infection. **(a)** Non-metric multidimensional scaling (NMDS) ordination based on Bray–Curtis dissimilarity, showing no significant separation between the archaeal communities of HRS and infected IRS rhizosphere soils (PERMANOVA, *R*^2^ = 0.087, *p* = 0.161). Statistical significance was assessed by PERMANOVA (9,999 permutations, Bray–Curtis), with *R*^2^ and *p* value indicated. **(b)** Boxplot comparing the Chao1 richness index of archaeal communities between HRS and IRS groups. No significant difference was observed (two-tailed Wilcoxon rank-sum test, *p* = 0.166). **(c)** Boxplot comparing the Shannon diversity index of archaeal communities between HRS and IRS groups. No significant difference was observed (two-tailed Wilcoxon rank-sum test, *p* = 0.436). **(d)** Bar chart showing the relative abundance of dominant archaeal phyla in HRS and IRS samples. No phylum exhibited a significant difference in abundance between the two groups. **(e)** Bar chart showing the relative abundance of the top archaeal genera in HRS and IRS samples. Several genera, including *Methanolobus*, *Methanocella*, and *Thermococcus*, showed significant differences in relative abundance (two-tailed Wilcoxon rank-sum test, *p* values as indicated). Despite these genus-level shifts, the overall community structure remained stable. HRS, healthy rhizosphere soil; IRS, infected rhizosphere soil; NMDS, non-metric multidimensional scaling; PERMANOVA, permutational multivariate analysis of variance.
